# Identification of differentially expressed lncRNAs and mRNAs in luminal-B breast cancer by RNA-sequencing

**DOI:** 10.1186/s12885-019-6395-5

**Published:** 2019-12-03

**Authors:** Cheng-Liang Yuan, Xiang-Mei Jiang, Ying Yi, Jian-Fei E, Nai-Dan Zhang, Xue Luo, Ning Zou, Wei Wei, Ying-Ying Liu

**Affiliations:** 1 Department of Clinical Laboratory, People’s Hospital of Deyang City, No. 173, Taishan North Road, Jingyang District, Deyang, 618000 Sichuan China; 2 Department of Breast Surgery, People’s Hospital of Deyang City, Deyang, China; 3 Department of Science and Education, People’s Hospital of Deyang City, Deyang, China

**Keywords:** Luminal-B breast cancer, mRNA, Long non-coding RNA (lncRNA), RNA-sequencing

## Abstract

**Background:**

Luminal B cancers show much worse outcomes compared to luminal A. This present study aims to screen key lncRNAs and mRNAs correlated with luminal-B breast cancer.

**Methods:**

Luminal-B breast cancer tissue samples and adjacent tissue samples were obtained from 4 patients with luminal-B breast cancer. To obtain differentially expressed mRNAs (DEmRNAs) and lncRNAs (DElncRNAs) between luminal-B breast cancer tumor tissues and adjacent tissues, RNA-sequencing and bioinformatics analysis were performed. Functional annotation of DEmRNAs and protein-protein interaction networks (PPI) construction were performed. DEmRNAs transcribed within a 100 kb window up- or down-stream of DElncRNAs were searched, which were defined as *cis* nearby-targeted DEmRNAs of DElncRNAs. DElncRNA-DEmRNA co-expression networks were performed. The mRNA and lncRNA expression profiles were downloaded from The Cancer Genome Atlas (TCGA) database to validate the expression patterns of selected DEmRNAs and DElncRNAs.

**Results:**

A total of 1178 DEmRNAs and 273 DElncRNAs between luminal-B breast cancer tumor tissues and adjacent tissues were obtained. Hematopoietic cell lineage, Cytokine-cytokine receptor interaction, Cell adhesion molecules (CAMs) and Primary immunodeficiency were significantly enriched KEGG pathways in luminal-B breast cancer. FN1, EGFR, JAK3, TUBB3 and PTPRC were five hub proteins of the PPI networks. A total of 99 DElncRNAs-nearby-targeted DEmRNA pairs and 1878 DElncRNA-DEmRNA co-expression pairs were obtained. Gene expression results validated in TCGA database were consistent with our RNA-sequencing results, generally.

**Conclusion:**

This study determined key genes and lncRNAs involved in luminal-B breast cancer, which expected to present a new avenue for the diagnosis and treatment of luminal-B breast cancer.

## Background

Breast cancer is the leading cause of cancer-related death in women, both overall and in less developed countries (1). It is a heterogeneous disease with regard to molecular alterations, cellular composition, and clinical outcome, both between tumor subtypes and within a single tumor, which were commonly defined by gene expression profiling as four main subtypes including luminal A, luminal B, HER-2 enriched and basal-like, (2–4). Luminal B breast cancer is unique with regard to somatic point mutations, the profile of gene copy number alterations (CNAs), and DNA methylation (5). Expression profiles and gene sets, with prognostic, predictive functions, or both for patients with breast cancer, have been identified in multiple studies (6). Although both luminal-A and luminal-B breast cancers are ER-positive, luminal-B cancers showed worse outcomes as compared to luminal-A cancers (7, 8). Therefore, it is urgent to discover novel biomarkers with prognostic and predictive functions for luminal B breast cancer that can be therapeutically targeted.

With advances in high-throughput technology, it is discovered that human transcriptome mainly consists of non-coding RNAs (ncRNAs) with limited or no protein-coding capacity (9, 10). Long non-coding RNAs (lncRNAs), with over 200 nucleotides base long, attracts more attention and has been widely linked with various diseases, including cancers (11–14). The lncRNAs exert momentous roles in multiple cellular processes at transcriptional and post-transcriptional regulation level through transcriptional interference and histone modifications (15, 16). The higher expression of SPRY4-IT1 was reported to modulate apoptosis and invasion in melanoma (17). LncRNA UCA1a (CUDR) may promote proliferation and tumorigenesis in human bladder cancer (18).

In this study, differentially expressed lncRNAs (DElncRNA) and mRNAs (DEmRNAs) in tumor tissues of patients with luminal-B breast cancer were identified by RNA-sequencing. Subsequently, protein-protein interaction (PPI) networks of DEmRNAs were conducted. Identification of *cis* nearby-targeted DEmRNAs of DElncRNAs and construction of DElncRNA-DEmRNA co-expression networks were performed. In this light, we expect this study could represent a new avenue to improve the understanding of the pathogenesis and be helpful for treatment of luminal B breast cancer.

## Methods

### Patients and samples

Luminal-B breast cancer tissue samples and adjacent tissue samples were obtained from 4 patients with luminal-B breast cancer in People’s Hospital of Deyang City, which were free of treatment. The detailed characteristics of patients are displayed in Table 1. Written informed consent about the use of these samples was obtained from each patient. All procedures performed in this study was in accordance with the ethical standards of the ethics committee of People’s Hospital of Deyang City (2017–045) and with the 1964 Helsinki declaration and its later amendments or comparable ethical standards.

**Table 1:** Patient characteristics

Index	Age Range	TNM stage			ER	PR	HER-2
		T	N	M			
Case 1	46–56	3	0	0	30%	10%	–
Case 2		2	1	0	80%	60%	+
Case 3		2	1	0	40%	30%	–
Case 4		2	1	0	40%	20%	–

*TNM* stage Tumor-node-metastasis stage, *ER* ertrogen receptor, *PR* progestrone receptor, *HER*-2 human epidermal growth factor receptor-2

### RNA isolation and sequencing

According to the manufacturer’s protocol, RNA was extracted with PAXgene blood RNA kit (PreAnalytiX GmbH, Hombrechtikon, CH, Switzerland). With Agilent 2100 Bioanalyzer (Agilent RNA 6000 Nano Kit), the concentration, integrity and RNA integrity number (RIN) values of RNA were assessed. Sequencing was performed based on the Illumina Hiseq X-ten platform (Illumina, Inc., San Diego, CA, USA) with PE150 bp sequencing mode. The sequencing was done with paired-ends and 10G depth. With Base Calling version 0.11.4 (http://www.bioinformatics.babraham.ac.uk/projects/fastqc/), the FASTQ sequence data were acquired from the RNA-sequencing data. Read QC tool in FastQC version 0.11.4 (http://www.bioinformatics.babraham.ac.uk/projects/fastqc/) was used for the quality control of FASTQ data with Q > 30. Trimming of raw data was performed with cutadapt version 1.16 (http://cutadapt.readthedocs.io). Reads with low quality (adaptor sequences, sequences with a quality score < 20, and sequences with an N base rate of raw reads > 10%) were removed to obtain the clean reads.

### Identification of DEmRNAs and DElncRNAs

In order to align the clean reads with the human reference genome, Ensemble GRCh38.p7 (ftp://ftp.ncbi.nlm.nih.gov/genomes/Homo_sapiens), HISAT2 version 2.1.0 (https://ccb.jhu.edu/software/hisat2/index.shtml) was applied. Expression of mRNAs and lncRNAs was normalized and outputted with StringTie version 1.3.3b (http://ccb.jhu.edu/software/stringtie/). Fragments per Kilobase of exon per million fragments mapped (FPKM) of lncRNAs and mRNAs were calculated with StringTie. With edgeR version 3.24 (http://www.bioconductor.org/packages/release/bioc/html/edgeR.html), both DEmRNAs and DElncRNAs were obtained with |log2FC| > 1 and *p*-value < 0.05. By using R package “pheatmap”, hierarchical clustering analysis of DElncRNAs and DEmRNAs were conducted.

### Functional annotation of DEmRNAs

Functional annotation, including Gene Ontology (GO) function and Kyoto Encyclopedia of Genes and Genomes (KEGG) pathway enrichment analyses was performed with Metascape (http://metascape.org/gp/index.html). A value of *p* < 0.05 was set as the cut-off for significance.

### Protein-protein interaction (PPI) networks construction

Top 100 up- and down-regulated DEmRNAs were scanned with the BioGrid (http://www.uniprot.org/database/DB-0184). Then, PPI networks were visualized with Cytoscape software (version 3.5.0, http://www.cytoscape.org).

### *Cis* nearby-targeted DEmRNAs of the DElncRNAs

DEmRNAs transcribed within a 100-kb window upstream or downstream of DElncRNAs were searched, which were defined as *cis* nearby-targeted DEmRNAs of DElncRNAs, to obtain the targeted DEmRNAs of DElncRNAs with *cis*-regulatory effects. The networks were visualized by Cytoscape software. Functional annotation of the *cis* nearby-targeted DEmRNAs of the DElncRNAs was performed with Metascape. A value of *p* < 0.05 was set as the cut-off for significance.

### DElncRNA-DEmRNA co-expression networks

To further examine the potential roles of DElncRNAs and DEmRNAs in luminal-B breast cancer, the DElncRNA-DEmRNA co-expression networks were constructed. DElncRNA-DEmRNA pairs with an absolute value of PCC > 0.95 and *p* < 0.01 were defined as co-expressed DElncRNA-DEmRNA pairs. By using Cytoscape, the co-expressed DElncRNA-DEmRNA networks were visualized. Functional annotation of the DEmRNAs co-expressed with DElncRNAs was performed with Metascape. A value of *p* < 0.05 was set as the cut-off for significance.

### Validation in the Cancer genome atlas (TCGA) database

The mRNA and lncRNA expression profiles of 171 patients with luminal B breast cancer and 59 normal tissues were downloaded from TCGA database to validate the expression patterns of selected DEmRNAs and DElncRNAs.

## Results

### DEmRNAs and DElncRNAs between luminal-B breast cancer tumor tissues and adjacent tissues

**Fig. 1:**
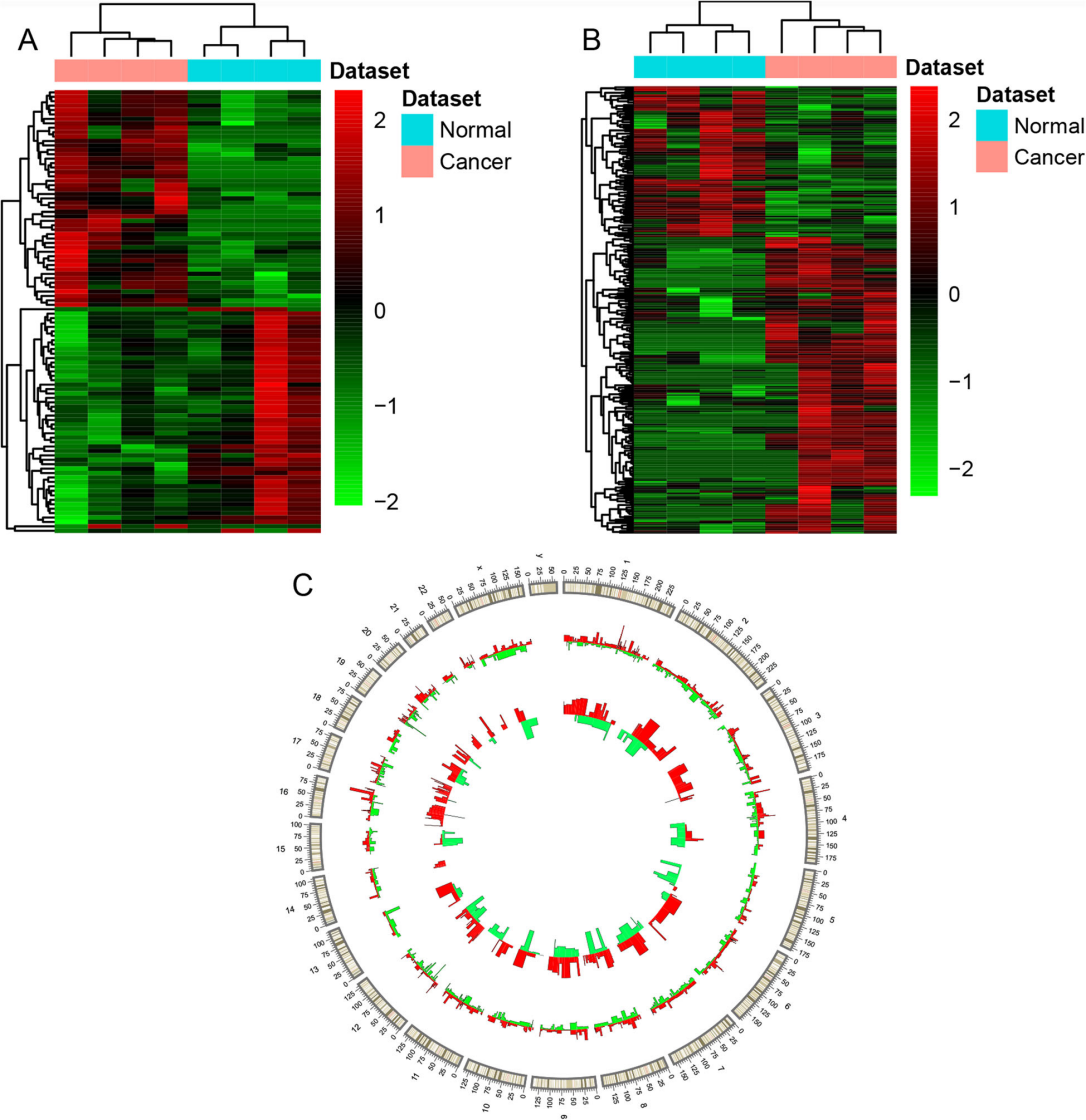
DElncRNAs and DEmRNAs between luminal-B breast cancer tumor tissues and adjacent tissues. **a** and **b** displayed hierarchical clustering results of top 100 DEmRNAs and DElncRNAs between luminal-B breast cancer tumor tissues and adjacent tissues, respectively. Row and column represented DEmRNAs/DElncRNAs and tissue samples, respectively. The color scale represented the expression levels. **c** displayed distribution of DElncRNAs and DEmRNAs on chromosomes. The outer layer cycle was the chromosome map of the human genome hg19 (GRCh37). The larger inner layer and smaller inner layer represented the distribution of DEmRNAs and DElncRNAs on different chromosome, respectively. The red and green color represented the up- and down-regulated

A total of 1178 DEmRNAs (666 up-regulated and 512 down-regulated DEmRNAs) and 273 DElncRNAs (181 up-regulated and 92 down-regulated DElncRNAs) were obtained. The top 10 up- and down-regulated DEmRNAs and DElncRNAs were exhibited in Table 2 and Table 3, respectively. Hierarchical clustering analysis of top 100 10 up- and down-regulated DEmRNAs and DElncRNAs was showed in Fig. 1a and Fig. 1b, respectively. Furthermore, the distribution of DElncRNAs and DEmRNAs on all chromosomes was showed in Fig. 1c. The raw-data have been uploaded to Gene Expression Omnibus (GEO) (GSE139274, https://www.ncbi.nlm.nih.gov/geo/query/acc.cgi?acc=GSE139274).

**Table 2:** Top 10 up- and down-regulated DEmRNAs between luminal-B breast cancer tumor tissues compared with adjacent tissues

ID	Symbol	log_2_FC	*p*-value	Regulation
ENSG00000143556	S100A7	8.127250867	7.08E-10	up
ENSG00000171951	SCG2	4.46199863	7.47E-09	up
ENSG00000163993	S100P	5.865266947	2.43E-08	up
ENSG00000169245	CXCL10	5.755282864	2.57E-08	up
ENSG00000188404	SELL	4.205586942	2.63E-08	up
ENSG00000180549	FUT7	3.310463733	3.42E-08	up
ENSG00000138755	CXCL9	4.319459635	4.19E-08	up
ENSG00000099953	MMP11	4.120231544	1.44E-07	up
ENSG00000184937	WT1	2.831855381	2.43E-07	up
ENSG00000143546	S100A8	5.625786123	3.05E-07	up
ENSG00000269711	AC008763.3	−8.573808999	3.62E-11	down
ENSG00000272414	FAM47E-STBD1	−4.255105776	3.94E-07	down
ENSG00000109846	CRYAB	−2.409617655	1.98E-06	down
ENSG00000109107	ALDOC	−2.539792463	3.92E-06	down
ENSG00000134548	SPX	−7.064583075	5.21E-06	down
ENSG00000135447	PPP1R1A	−4.272501282	8.38E-06	down
ENSG00000120049	KCNIP2	−3.142698521	1.76E-05	down
ENSG00000270181	BIVM-ERCC5	−5.716264875	1.93E-05	down
ENSG00000162433	AK4	−1.720478242	1.94E-05	down
ENSG00000159387	IRX6	−4.343462068	3.97E-05	down

DEmRNAs, differentially expressed mRNAs. FC, fold change

**Table 3:** Top 10 up- and down-regulated DElncRNAs between luminal-B breast cancer tumor tissues compared with adjacent tissues

ID	Symbol	log_2_FC	*p*-value	Regulation
ENSG00000235123	DSCAM-AS1	6.99539501	2.71E-06	up
ENSG00000273445	AC133644.2	7.081588171	3.65E-06	up
ENSG00000261039	LINC02544	7.125899953	1.29E-05	up
ENSG00000225783	MIAT	2.369853869	3.75E-05	up
ENSG00000224950	AL390066.1	2.333212245	7.65E-05	up
ENSG00000270120	AC007728.3	5.314205083	8.62E-05	up
ENSG00000279930	AL032819.2	5.526214467	1.62E-04	up
ENSG00000247774	PCED1B-AS1	1.82134551	1.78E-04	up
ENSG00000261218	AC099524.1	4.377268771	2.60E-04	up
ENSG00000234261	AL138720.1	4.488627977	2.87E-04	up
ENSG00000253434	LINC02237	−6.553462635	1.55E-04	down
ENSG00000250961	AC025470.2	−5.149166872	1.76E-04	down
ENSG00000236333	TRHDE-AS1	−4.862423419	4.38E-04	down
ENSG00000261441	AC124068.2	−4.394333958	7.43E-04	down
ENSG00000261888	AC144831.1	−3.00198307	1.98E-03	down
ENSG00000251660	AC007036.3	−2.997221226	2.05E-03	down
ENSG00000260947	AL356489.2	−3.611436753	2.33E-03	down
ENSG00000235033	AL590999.1	−2.980398474	2.69E-03	down
ENSG00000230333	AC004160.1	−3.679435378	2.71E-03	down
ENSG00000272701	MESTIT1	−3.528818654	2.85E-03	down

DElncRNAs, differentially expressed lncRNAs. FC, fold change

### Functional annotation

**Fig. 2:**
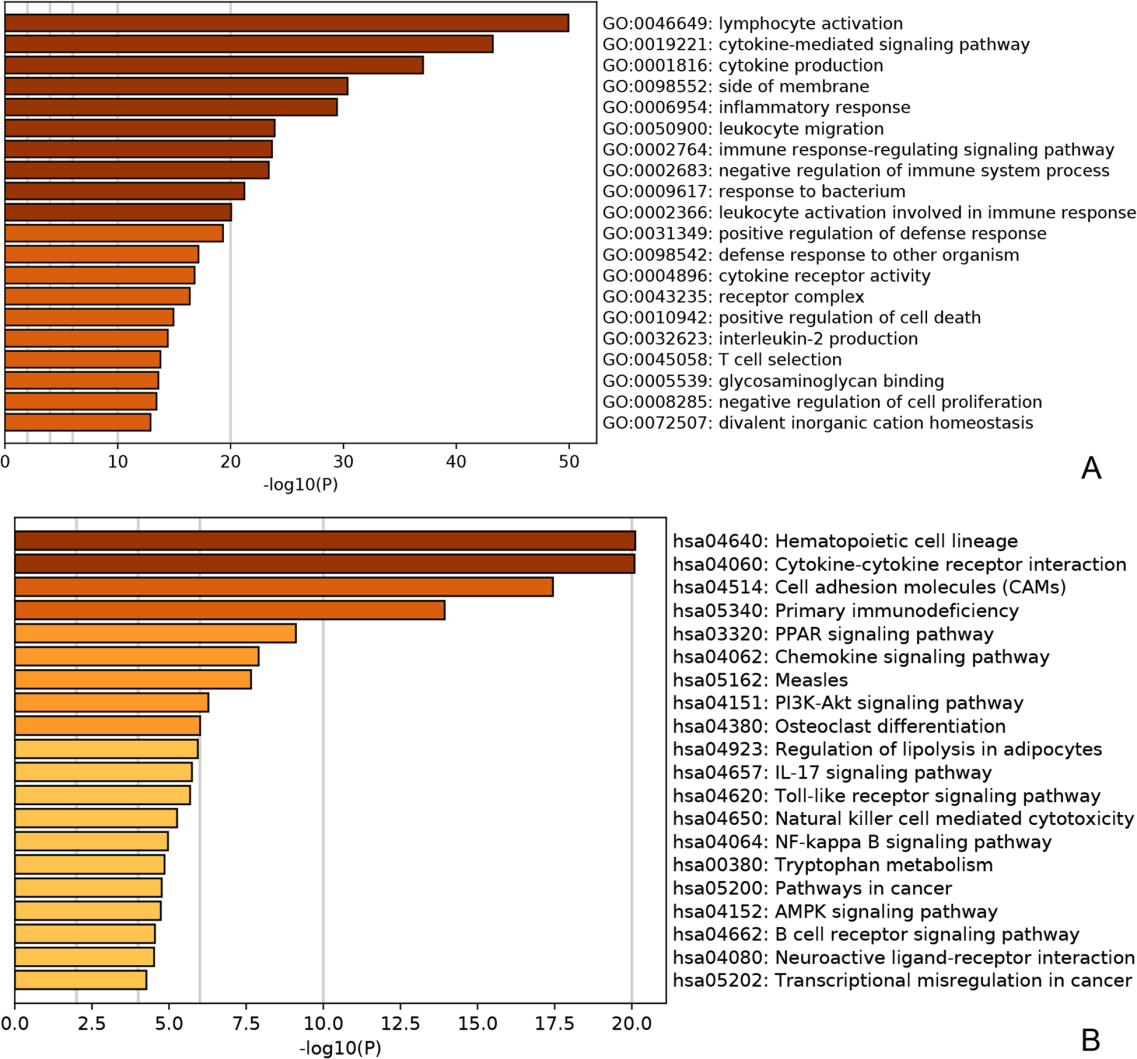
Significantly enriched GO (**a**) terms and KEGG (**b**) pathways of DEmRNAs between luminal-B breast cancer tumor tissues and adjacent tissues. The x-axis represented -lg *p*-value and the y-axis shows GO terms or KEGG pathways

Lymphocyte activation (*p* = 1.14E-50), cytokine-mediated signaling pathway (*p* = 5.74E-44) and cytokine production (*p* = 8.78E-38) were significantly enriched GO terms in luminal-B breast cancer (Fig. 2a). Hematopoietic cell lineage (*p* = 7.75E-21), Cytokine-cytokine receptor interaction (*p* = 8.19E-21), Cell adhesion molecules (CAMs) (*p* = 3.61E-18) and Primary immunodeficiency (*p* = 1.17E-14) were significantly enriched KEGG pathways in luminal-B breast cancer (Fig. 2b).

### Protein-protein interaction (PPI) networks

**Fig. 3:**
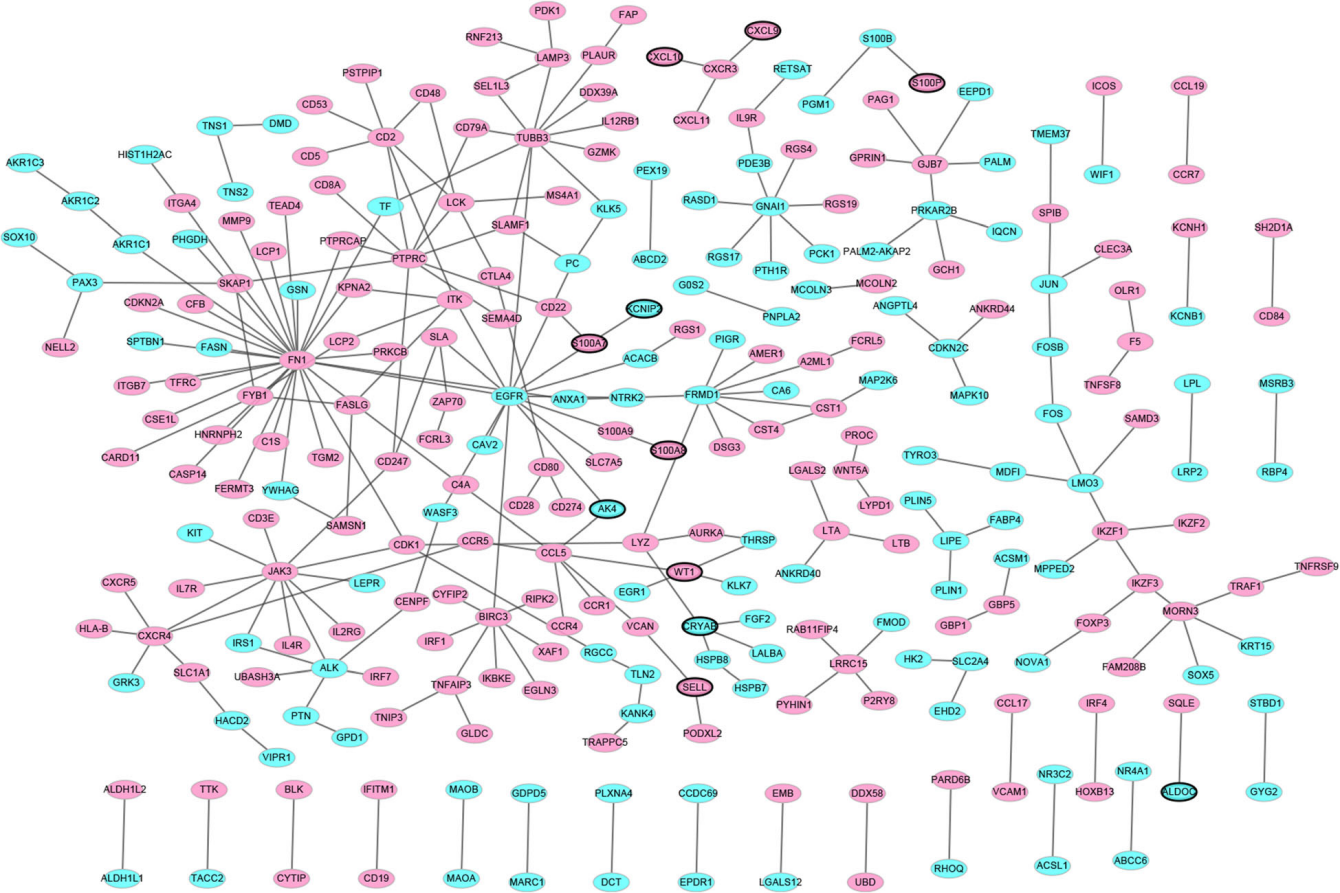
Protein-protein interaction (PPI) networks. The red and blue ellipses represented proteins encoded by up- and down-regulated DEmRNAs between luminal-B breast cancer tumor tissues and adjacent tissues. Ellipses with black border were DEmRNAs derived from top 10 down- and up-regulated DEmRNAs between luminal-B breast cancer tumor tissues and adjacent tissues

The PPI networks included 281 nodes and 263 edges. FN1 (degree = 28), EGFR (degree = 14), JAK3 (degree = 11), TUBB3 (degree = 11) and PTPRC (degree = 10) were five hub proteins of the PPI networks (Fig. 3).

### *Cis*-nearby-targeted DEmRNAs of DElncRNAs

**Fig. 4:**
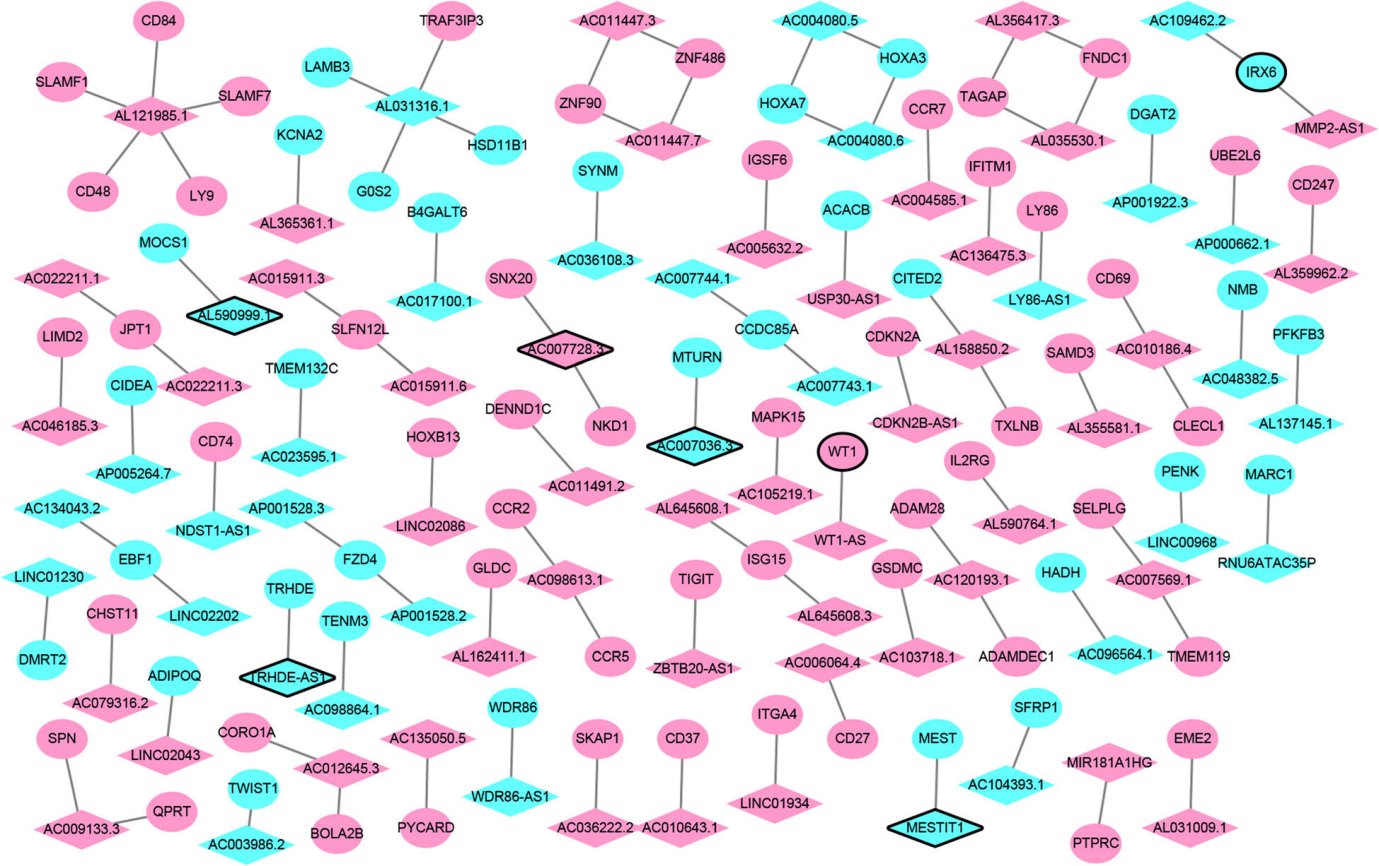
DElncRNA-nearby DEmRNA interaction networks. The inverted triangles and ellipses represent DElncRNAs and their nearby DEmRNAs between luminal-B breast cancer tumor tissues and adjacent tissues, respectively. Red and blue color represent up- and down-regulation in luminal-B breast cancer tumor tissues compared to adjacent tissues, respectively. Inverted triangles and ellipses with black border were DElncRNAs/DEmRNAs derived from top 10 up- and down-regulated DElncRNAs/DEmRNAs

A total of 99 DElncRNAs-nearby-targeted DEmRNA pairs, involving in 78 DElncRNAs and 86 DEmRNAs, were detected (Fig. 4). Top two DElncRNAs with most nearby DEmRNAs were AL121985.1 and AL031316.1, which owned 5 and 4 nearby DEmRNAs, respectively. Regulation of cell adhesion (*p* = 1.84E-13), regulation of cell-cell adhesion (*p* = 7.86E-12), cytokine binding (*p* = 3.41E-07), T cell selection (*p* = 7.75E-07) and negative regulation of secretion (*p* = 1.38E-06) were significantly enriched GO terms (Fig. 6a). Cell adhesion molecules (CAMs) (*p* = 1.72E-04), Cytokine-cytokine receptor interaction (*p* = 2.81E-03), Primary immunodeficiency (*p* = 7.71E-03), AMPK signaling pathway (*p* = 9.30E-03) and Wnt signaling pathway (*p* = 1.46E-02) were significantly enriched KEGG pathways (Fig. 6b).

### DElncRNA-DEmRNA co-expression networks

**Fig. 5:**
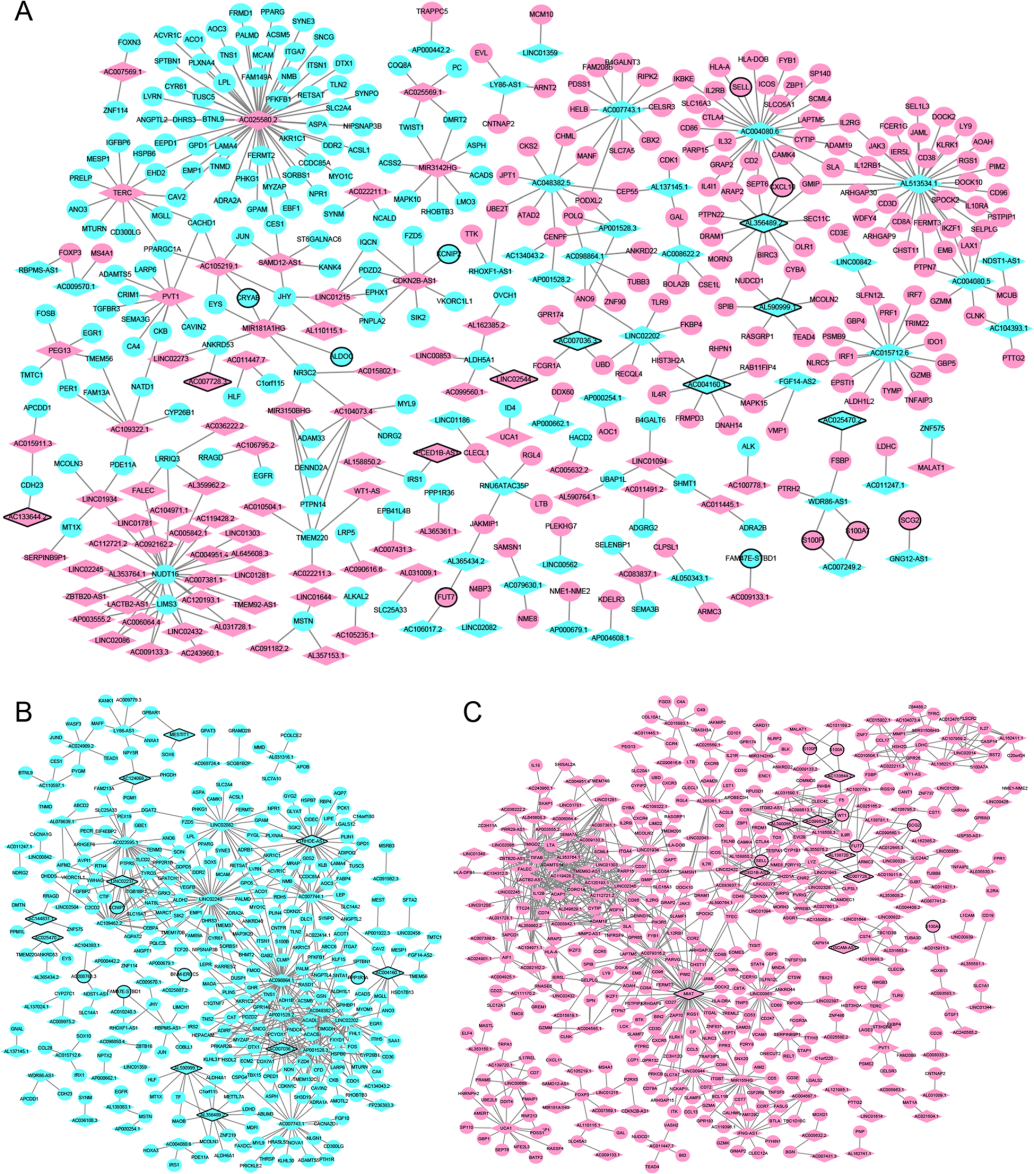
DElncRNA-DEmRNA co-expression networks. The inverted triangles and ellipses represent DElncRNAs and their nearby DEmRNAs between luminal-B breast cancer tumor tissues and adjacent tissues, respectively. Red and blue color represent up- and down-regulation in luminal-B breast cancer tumor tissues compared to adjacent tissues, respectively. Inverted triangles and ellipses with black border were DElncRNAs/DEmRNAs derived from top 10 up- and down-regulated DElncRNAs/DEmRNAs

A total of 1878 DElncRNA-DEmRNA co-expression pairs including 225 DElncRNAs and 737 DEmRNAs were obtained with an absolute value of PCC > 0.95 and *p* < 0.01 (Fig. 5).

**Fig. 6:**
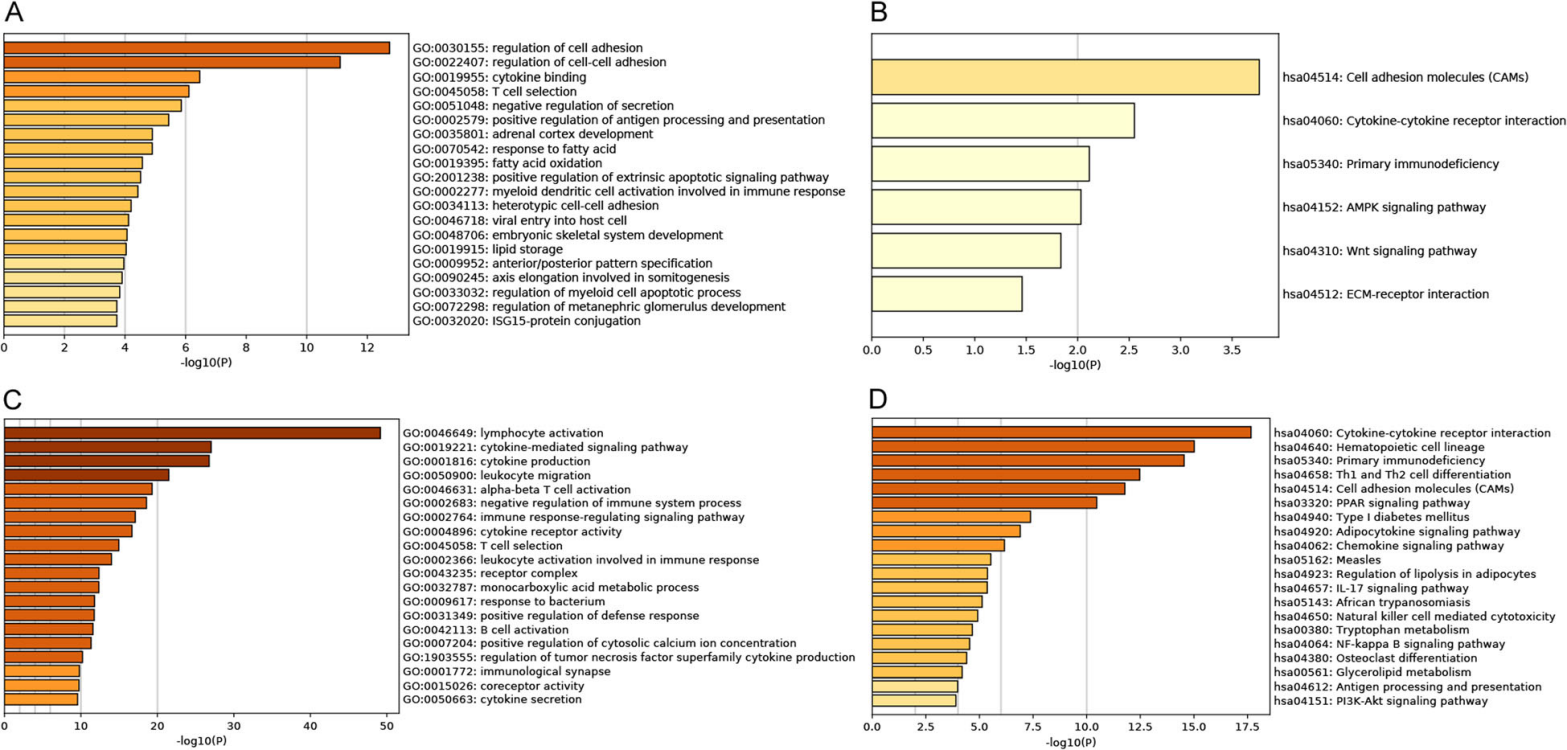
Significantly enriched GO terms and KEGG pathways of DElncRNA-nearby DEmRNAs (**a-b**) and DEmRNAs co-expressed with DElncRNAs (**c-d**). The x-axis represented -lg *p*-value and the y-axis shows GO terms or KEGG pathways

Lymphocyte activation (*p* = 7.08E-50), cytokine-mediated signaling pathway (*p* = 9.35E-28), cytokine production (*p* = 1.68E-27), leukocyte migration (*p* = 3.20E-22) and alpha-beta T cell activation (*p* = 4.89E-20) were significantly enriched GO terms (Fig. 6c). Cytokine-cytokine receptor interaction (*p* = 2.18E-18), Hematopoietic cell lineage (*p* = 9.66E-16), Primary immunodeficiency (*p* = 2.85E-15), Th1 and Th2 cell differentiation (*p* = 3.38E-13) and Cell adhesion molecules (CAMs) (*p* = 1.66E-12) were significantly enriched KEGG pathways (Fig. 6d).

### Validation in TCGA database

**Fig. 7:**
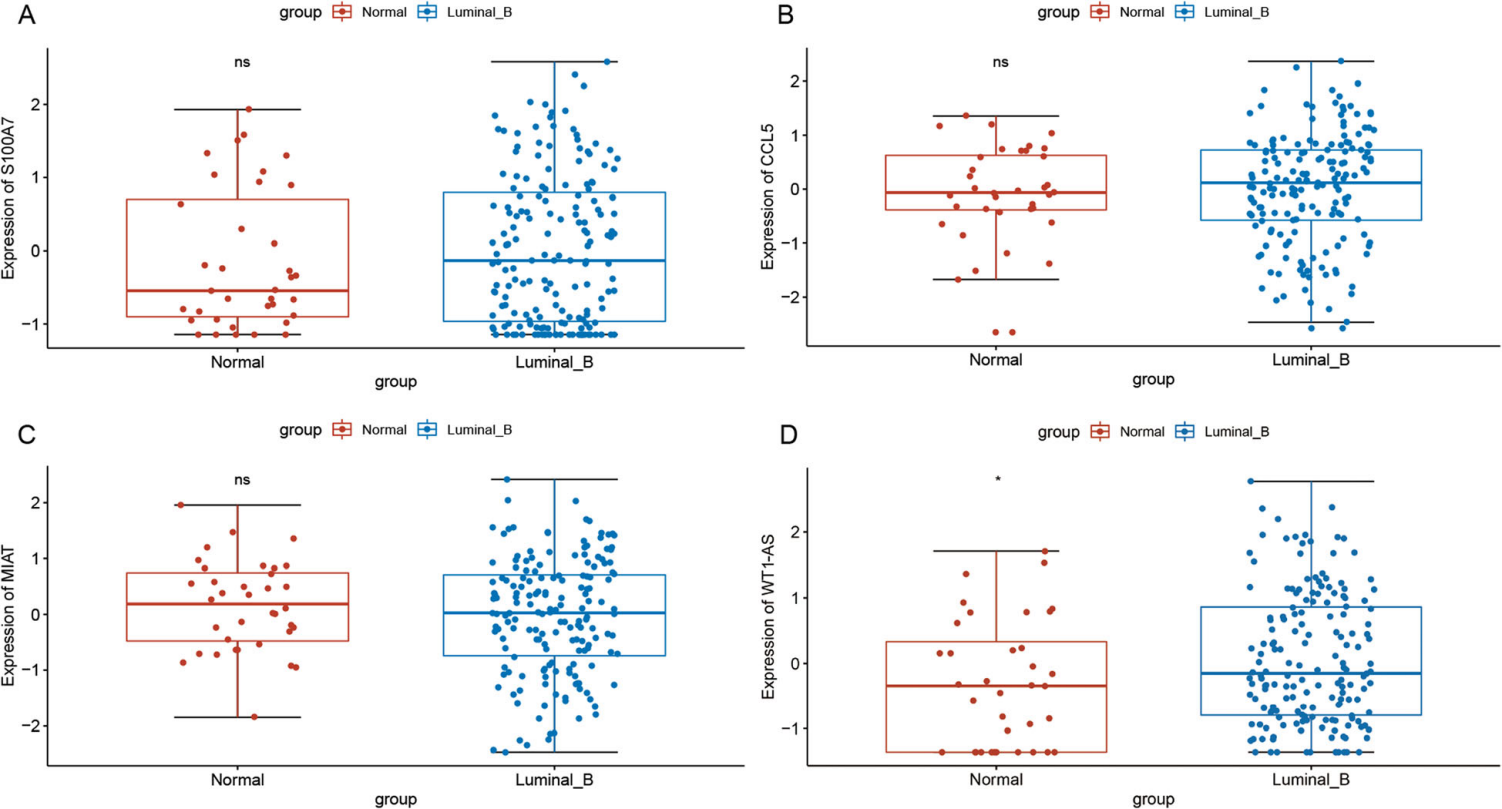
Validation in TCGA database. The x-axis shows luminal-B breast cancer tumor tissues and adjacent tissues, and the y-axis shows expression levels, respectively. A) S100A7; B) CCL5; C) MIAT; D) WT1-AS

With mRNA and lncRNA expression profiles downloaded from TCGA database, the expression patterns of four DEmRNAs, including S100A7, CCL5, MIAT and WT1-AS, were verified. As shown in Fig. 7, compared to normal controls, MIAT was down-regulated in luminal B breast cancer tumor tissues which were inconsistent with our results, while S100A7, CCL5 and WT1-AS were up-regulated in luminal B breast cancer tumor tissues which were consistent with our results.

## Discussion

Breast cancer, as the most common non-cutaneous type of cancer, is the leading cause of cancer-related mortality among female globally (19). As luminal-B cancers showed poorer prognosis as compared to luminal-A cancers, we performed this study and identified abundant DElncRNAs and DEmRNAs between luminal-B breast cancer tumor tissues and adjacent tissues.

S100A7 is a member of the S100 protein family, which have been associated with preinvasive ductal carcinoma in situ (DCIS) (20). During breast tumorigenesis and/or progression, several S100 s, including S100A2, S100A4 and S100A7, exhibit altered expression levels based on molecular analysis of breast tumors (21). Cancemi et al. suggested that S100A7 was involved in critical phases of the breast cancer growth and progression (22). Mayama et al. proposed that S100A7 was linked to an aggressive phenotype of ER-positive breast carcinoma, and was potent marker for distant metastasis of ER-positive breast cancer patients (23). In current study, S100A7 was the most significant up-regulated DEmRNAs in luminal B breast cancer tumor tissues, which may indicated that S100A7 exert momentous roles in luminal B breast cancer.

Metastasis-associated lung adenocarcinoma transcript 1 (MALAT1) is a highly conserved lncRNA, and its over-expression in multiple cancerous tissues has been linked to the proliferation and metastasis of tumor cells. It was first identified as being up-regulated in lung tumors, and a prognostic marker for metastasis and patient survival in non-small cell lung cancer (NSCLC), specifically in early stages of lung adenocarcinoma (24). Subsequently, MALAT1 was shown to be up-regulated in a broad spectrum of tumor types, such as endometrial stromal sarcoma and hepatocellular carcinomas (25, 26). Additional, it has been found that MALAT1 gene mutations frequently occurred in luminal-type breast tumors (27). Besides, MALAT1 was one of the top 10 up-regulated DElncRNAs in this study, and co-expressed with S100A7, which emphasized the critical role the MALAT1 in luminal B breast cancer and suggested that MALAT1 may involve in luminal B breast cancer by regulating S100A7.

Chemokines, small-molecular-weight cytokines involved in the physiological control of immune cell migrationare, were reported to perform a crucial function in breast cancer tumorigenesis and progression (19). Recent years, the chemokine C-C motif ligand 5 (CCL5), also known as RANTES, is a member of the CC subfamily, has been associated with aggressive breast cancer (28). Svensson et al. identified CCL2 and CCL5 as two therapeutic targets for estrogen-dependent breast cancer (29). Previous study suggested that endothelial cells (ECs) enhance endothelial-mesenchymal transition (EMT)-induced triple-negative breast cancer (TNBC) cell metastasis through PAI-1 and CCL5 signaling (30). Zhang et al. found that CCL5-mediated Th2 polarization of CD4^+^ T cells promotes metastasis in luminal breast cancer (31). In our analysis, CCL5 was significant up-regulated in luminal B breast cancer tumor tissues, which indicated the key role of CCL5 in luminal B breast cancer.

LncRNA myocardial infarction-associated transcript (MIAT) is primarily expressed in heart and fetal brain tissue (32). Dysregulated MIAT was first reported to correlated with myocardial infarction and involved in cardiac hypertrophy and diabetic cardiomyopathy (32–35). Recent studies suggested that MIAT promoted gastric cancer growth and metastasis by regulation of miR-141/DDX5 pathway, promoted proliferation and metastasis of non-small cell lung cancer via MMP9 activation, promoted hepatocellular carcinoma cells proliferation and invasion through sponging miR-214 (36–38). Luan et al. proposed that overexpression of MIAT was related to the TNM stage and lymphnode metastasis of breast cancer (39). MIAT was found to be overexpressed in both ER-positive breast cancer tissues and ER-positive breast cancer cell line MCF-7, and play a pivotal role in ER-positive breast cancer cell growth (40). Almnaseer-M et al. suggested that MIAT performs a critical function in breast tumorigenesis (41). MIAT was detected to be one of the top 10 up-regulated DElncRNAs, and co-expressed with CCL5. All these findings suggest that MIAT may involve in luminal B breast cancer by regulating the expression level of CCL5.

The Wilms’ tumor 1 (WT1) was first cloned in 1990 as a suppressor in Wilms’ tumor, which was located at chromosome 11p13 (42). WT1 gene mutations are linked with a subset of Wilm’s tumors, the most common pediatric renal cancer (43). Substantial evidence has linked WT1 with the pathogenesis of breast cancer. WT1 is linked to the progression of breast cancer, including migration, invasion and angiogenesis. Knockdown of WT1 was demonstrated to lead to mitochondrial damage and then inhibit malignant cell growth (44). Highly expressed WT1 was linked to poor prognosis of patients with breast cancer (45). Over expression of WT1 was detected in TNBC (46). In agreement with previous studies, the expression of WT1 was observed significant up-regulated in luminal B breast cancer tumor tissues in present study.

Wilms tumor 1 Antisense RNA (WT1-AS) is located upstream of the WT1 gene, and these two genes are bi-directionally transcribed from the same promoter region. Down-regulation of WT1-AS was related to a poorer prognosis in ovarian clear cell adenocarcinoma (47). Lv et al. suggested that WT1-AS promoted cell apoptosis in hepatocellular carcinoma (HCC) and may function as a tumor suppressor in HCC (48). It is reported that WT1-AS was significantly down-regulated in gastric cancers and may correlates with tumor progression (49). In addition, WT1-AS was detected to be was under-expressed in cervical carcinoma and suppress cervical cancer cell growth and aggressiveness (50, 51). In the current study, we found that WT1-AS was a DElncRNA and WT1 was a nearby-targeted DEmRNA of WT1-AS, which reminded us to explore the role of WT1-AS-WT1 in luminal B breast cancer.

## Conclusion

In conclusion, a total of 1178 DEmRNAs and 273 DElncRNAs between luminal-B breast cancer tumor tissues and adjacent tissues were obtained. We discussed and emphasized the importance role of three DElncRNA-DEmRNA pairs, including MALAT1-S100A7, MIAT-CCL5 and WT1-AS-WT1, involved in luminal B breast cancer, which expected to provide new insight into understanding the mechanism underlying pathogenesis of luminal B breast cancer. The small sample size was a limitation of our study. Although the validation results in TCGA database indicated that our RNA-sequencing results were generally reliable, larger cohorts of patients and further experimental validation studies are needed to conduct to verify this conclusion.

## Data Availability

The raw-data have been uploaded to Gene Expression Omnibus (GEO) (GSE139274, https://www.ncbi.nlm.nih.gov/geo/query/acc.cgi?acc=GSE139274) and is publicly available until October 2020 due to data confidentiality.

## Abbreviations

CAMs: Cell adhesion molecules
CCL5: Chemokine C-C motif ligand 5
CNAs: Copy number alterations
DCIS: Ductal carcinoma in situ
DElncRNA: Differentially expressed lncRNA
DEmRNA: Differentially expressed mRNA
ECs: Endothelial cells
EMT: Endothelial-mesenchymal transition
FPKM: Per million fragments mapped
GO: Gene Ontology
HCC: Hepatocellular carcinoma
KEGG: Kyoto Encyclopedia of Genes and Genomes
lncRNAs: Long non-coding RNAs
MALAT1: Metastasis-associated lung adenocarcinoma transcript 1
MIAT: Myocardial infarction-associated transcript
ncRNAs: Non-coding RNAs
NSCLC: Non-small cell lung cancer
PCC: Pearson’s correlation coefficient
PPI: Protein-protein interaction network
RIN: RNA integrity number
TNBC: Triple-negative breast cancer
WT1: Wilms’ tumor 1
WT1-AS: Wilms tumor 1 Antisense RNA

## References

[1] Torre LA, Bray F, Siegel RL, Ferlay J, Lortet-Tieulent J, Jemal A (2015). Global cancer statistics, 2012. CA Cancer J Clin.

[2] Parker JS, Michael M, Cheang MCU, Samuel L, David V, Tammi V (2009). Supervised risk predictor of breast cancer based on intrinsic subtypes. J Clin Oncol.

[3] Kornelia P (2011). Heterogeneity in breast cancer. J Clin Investig.

[4] Untch M, Gerber B, Harbeck N, Jackisch C, Marschner N, Mobus V, et al. 13th st. Gallen international breast cancer conference 2013: primary therapy of early breast cancer evidence, controversies, consensus - opinion of a german team of experts (zurich 2013). Breast care (Basel, Switzerland). 2013;8(3):221–9. 10.1159/000351692 PMC3728634 24415975

[5] Weinstein JN, Collisson EA, Mills GB, Shaw KRM, Ozenberger BA, Kyle E (2015). The Cancer genome atlas Pan-Cancer analysis project. Chin J Lung Cancer.

[6] Cheng F, Oh DS, Lodewyk W, Britta W, Nuyten DSA, Nobel AB (2006). Concordance among gene-expression-based predictors for breast cancer. N Engl J Med.

[7] SøRlie T, ., Perou CM, Tibshirani R, ., Aas T, ., Geisler S, ., Johnsen H, ., et al. Gene expression patterns of breast carcinomas distinguish tumor subclasses with clinical implications. Proc Natl Acad Sci 2001. 10.1073/pnas.191367098 PMC58566 11553815

[8] Christos S, Lajos P (2009). Gene-expression signatures in breast cancer. N Engl J Med.

[9] Ponting CP, Oliver PL, Reik W (2009). Evolution and functions of long noncoding RNAs. Cell..

[10] Wilusz JE, Hongjae S, Spector DL (2009). Long noncoding RNAs: functional surprises from the RNA world. Genes Dev.

[11] Wapinski O, Chang HY (2011). Long noncoding RNAs and human disease. Trends Cell Biol.

[12] Francesco C, Akira W, Luca Q, Hui X, Larissa P, Abhijit P (2014). Identification of a long non-coding RNA as a novel biomarker and potential therapeutic target for metastatic prostate cancer. Oncotarget.

[13] Zhuang M, Gao W, Xu J, Wang P, Shu Y (2014). The long non-coding RNA H19-derived miR-675 modulates human gastric cancer cell proliferation by targeting tumor suppressor RUNX1. Biochem Biophys Res Commun.

[14] Wang Y, Wu N, Liu J, Wu Z, Dong D (2015). FusionCancer: a database of cancer fusion genes derived from RNA-seq data. Diagn Pathol.

[15] Mercer TR, Dinger ME, Mattick JS (2009). Long non-coding RNAs: insights into functions. Nat Rev Genet.

[16] Sun L, Li Y, Yang B (2016). Downregulated long non-coding RNA MEG3 in breast cancer regulates proliferation, migration and invasion by depending on p53’s transcriptional activity. Biochem Biophys Res Commun.

[17] Khaitan D, Dinger ME, Mazar J, Crawford J, Smith MA, Mattick JS (2011). The melanoma-upregulated long noncoding RNA SPRY4-IT1 modulates apoptosis and invasion. Cancer Res.

[18] Wang Y, Chen W, Yang C, Wu W, Wu S, Qin X (2012). Long non-coding RNA UCA1a(CUDR) promotes proliferation and tumorigenesis of bladder cancer. Int J Oncol.

[19] Marco VV, Wendy X, Pestell RG (2014). The potential to target CCL5/CCR5 in breast cancer. Expert Opin Ther Targets.

[20] Liu H, Wang L, Wang X, Cao Z, Yang Q, Zhang K (2013). S100A7 enhances invasion of human breast cancer MDA-MB-468 cells through activation of nuclear factor-kappaB signaling. World J Surg Oncol.

[21] Emberley ED, Murphy LC, Watson PH (2004). S100A7 and the progression of breast cancer. Breast Cancer Res.

[22] Cancemi P, Di Cara G, Albanese NN, Costantini F, Marabeti MR, Musso R (2012). Differential occurrence of S100A7 in breast cancer tissues: a proteomic-based investigation. Proteomics Clin Appl.

[23] Mayama A, Takagi K, Suzuki H, Sato A, Onodera Y, Miki Y (2018). OLFM4, LY6D and S100A7 as potent markers for distant metastasis in estrogen receptor-positive breast carcinoma. Cancer Sci.

[24] Ji P, Diederichs S, Wang W, Boing S, Metzger R, Schneider PM (2003). MALAT-1, a novel noncoding RNA, and thymosin beta4 predict metastasis and survival in early-stage non-small cell lung cancer. Oncogene..

[25] Yamada K, Kano J, Tsunoda H, Yoshikawa H, Okubo C, Ishiyama T (2006). Phenotypic characterization of endometrial stromal sarcoma of the uterus. Cancer Sci.

[26] Lin R, Maeda S, Liu C, Karin M, Edgington TS (2007). A large noncoding RNA is a marker for murine hepatocellular carcinomas and a spectrum of human carcinomas. Oncogene..

[27] Ellis MJ, Ding L, Shen D, Luo J, Suman VJ, Wallis JW (2012). Whole-genome analysis informs breast cancer response to aromatase inhibition. Nature..

[28] Ayesha K, Joy W, Ilaria F, Chaofeng M, Junhua M, Zhizhou Y, et al. Recent Advances in Discovering the Role of CCL5 in Metastatic Breast Cancer. Mini Reviews in Medicinal Chemistry. 2016;15(13). 10.2174/138955751513150923094709 PMC4968951 26420723

[29] Svensson S, Abrahamsson A, Rodriguez GV, Olsson AK, Jensen L, Cao Y (2015). CCL2 and CCL5 are novel therapeutic targets for estrogen-dependent breast Cancer. Clin Cancer Res.

[30] Zhang Wenwen, Xu Jing, Fang Hehui, Tang Lin, Chen Weiwei, Sun Qian, Zhang Qun, Yang Fang, Sun Zijia, Cao Lulu, Wang Yucai, Guan Xiaoxiang (2018). Endothelial cells promote triple-negative breast cancer cell metastasisviaPAI-1 and CCL5 signaling. The FASEB Journal.

[31] Qianfei Z, Jilong Q, Lin Z, Lei G, Bing Z, Yan Z (2015). CCL5-mediated Th2 immune polarization promotes metastasis in luminal breast Cancer. Cancer Res.

[32] Zhu XH, Yuan YX, Rao SL, Wang P (2016). LncRNA MIAT enhances cardiac hypertrophy partly through sponging miR-150. Euro Rev Med Pharmacol Sci.

[33] Zhou X, Zhang W, Jin M, Chen J, Xu W, Kong X (2017). lncRNA MIAT functions as a competing endogenous RNA to upregulate DAPK2 by sponging miR-22-3p in diabetic cardiomyopathy. Cell Death Dis.

[34] Li Y, Wang J, Sun L, Zhu S (2017). LncRNA myocardial infarction-associated transcript (MIAT) contributed to cardiac hypertrophy by regulating TLR4 via miR-93. Eur J Pharmacol.

[35] Ishii N, Ozaki K, Sato H, Mizuno H, Saito S, Takahashi A (2006). Identification of a novel non-coding RNA, MIAT , that confers risk of myocardial infarction. J Hum Genet.

[36] Sha M, Lin M, Wang J, Ye J, Xu J, Xu N (2018). Long non-coding RNA MIAT promotes gastric cancer growth and metastasis through regulation of miR-141/DDX5 pathway. Journal of experimental & clinical cancer research : CR.

[37] Lai I, Yang CA, Lin PC, Chan WL, Lee YT, Yen JC (2017). Long noncoding RNA MIAT promotes non-small cell lung cancer proliferation and metastasis through MMP9 activation. Oncotarget..

[38] Huang X, Gao Y, Qin J, Lu S (2018). lncRNA MIAT promotes proliferation and invasion of HCC cells via sponging miR-214. Am J Physiol Gastrointest Liver Physiol.

[39] Luan T, Zhang X, Wang S, Song Y, Zhou S, Lin J (2017). Long non-coding RNA MIAT promotes breast cancer progression and functions as ceRNA to regulate DUSP7 expression by sponging miR-155-5p. Oncotarget.

[40] Li Y, Jiang B, Wu X, Huang Q, Chen W, Zhu H (2018). Long non-coding RNA MIAT is estrogen-responsive and promotes estrogen-induced proliferation in ER-positive breast cancer cells. Biochem Biophys Res Commun.

[41] Almnaseer ZA, Mourtada-Maarabouni M. Long noncoding RNA MIAT regulates apoptosis and the apoptotic response to chemotherapeutic agents in breast cancer cell lines. Biosci Rep. 2018;38(4). 10.1042/BSR20180704 PMC6435567 29914974

[42] Call KM, Glaser T, Ito CY, Buckler AJ, Pelletier J, Haber DA (1990). Isolation and characterization of a zinc finger polypeptide gene at the human chromosome 11 Wilms' tumor locus. Cell.

[43] Silberstein GB, Van Horn K, Strickland P, Roberts CT, Daniel CW (1997). Altered expression of the WT1 wilms tumor suppressor gene in human breast cancer. Proc Natl Acad Sci U S A.

[44] Tatsumi N, Oji Y, Tsuji N, Tsuda A, Higashio M, Aoyagi S (2008). Wilms' tumor gene WT1-shRNA as a potent apoptosis-inducing agent for solid tumors. Int J Oncol.

[45] Yasuo M, Akiko A, Chiyomi E, Tetsuya T, Yasuhiro T, Hiroya T (2002). High expression of Wilms' tumor suppressor gene predicts poor prognosis in breast cancer patients. Clin Cancer Res.

[46] Xiao-Wei Q, Fan Z, Xin-Hua Y, Lin-Jun F, Yi Z, Yan L (2012). High Wilms' tumor 1 mRNA expression correlates with basal-like and ERBB2 molecular subtypes and poor prognosis of breast cancer. Oncol Rep.

[47] Kaneuchi M, Sasaki M, Tanaka Y, Shiina H, Yamada H, Yamamoto R (2005). WT1 and WT1-AS genes are inactivated by promoter methylation in ovarian clear cell adenocarcinoma. Cancer..

[48] Lv L, Chen G, Zhou J, Li J, Gong J (2015). WT1-AS promotes cell apoptosis in hepatocellular carcinoma through down-regulating of WT1. J Exp Clin Cancer Res : CR..

[49] Du T, Zhang B, Zhang S, Jiang X, Zheng P, Li J (2016). Decreased expression of long non-coding RNA WT1-AS promotes cell proliferation and invasion in gastric cancer. Biochim Biophys Acta.

[50] Cui L, Nai M, Zhang K, Li L, Li R (2019). lncRNA WT1-AS inhibits the aggressiveness of cervical cancer cell via regulating p53 expression via sponging miR-330-5p. Cancer Manag Res.

[51] Dai SG, Guo LL, Xia X, Pan Y (2019). Long non-coding RNA WT1-AS inhibits cell aggressiveness via miR-203a-5p/FOXN2 axis and is associated with prognosis in cervical cancer. Eur Rev Med Pharmacol Sci.

